# SARS-CoV-2 Entry Can Be Mimicked in *C. elegans* Expressing Human ACE2: A New Tool for Pharmacological Studies

**DOI:** 10.3390/v17101387

**Published:** 2025-10-18

**Authors:** Margherita Romeo, Sara Baroni, Maria Monica Barzago, Samuela Gambini, Ada De Luigi, Daniela Iaconis, Andrea Rosario Beccari, Maddalena Fratelli, Luisa Diomede

**Affiliations:** 1 Department of Molecular Biochemistry and Pharmacology, Istituto di Ricerche Farmacologiche Mario Negri IRCCS, 20156 Milano, Italy; margherita.romeo@marionegri.it (M.R.); sara.baroni@marionegri.it (S.B.); mariamonica.barzago@marionegri.it (M.M.B.); gambinisamuela@gmail.com (S.G.); ada.deluigi@marionegri.it (A.D.L.); maddalena.fratelli@marionegri.it (M.F.); 2 EXSCALATE, Dompé Farmaceutici SpA, Via Tommaso De Amicis, 95, 80131 Napoli, Italy; daniela.iaconis@exscalate.eu (D.I.); andrea.beccari@dompe.com (A.R.B.)

**Keywords:** COVID-19, SARS-CoV-2, receptor binding domain, angiotensin-converting enzyme 2, *C. elegans*, pseudo infection, raloxifene

## Abstract

Testing medical countermeasures for SARS-CoV-2 transmission using vertebrates can be hindered by legislation regulating animal experimentation, high costs, and ethical concerns. To overcome these challenges, we propose a new *Caenorhabditis elegans* strain that constitutively expresses the human angiotensin-converting enzyme 2 receptor (ACE2). This resulted in significant impairment of reproduction and a defect in pharyngeal function compared to wild-type (WT) worms. SARS-CoV-2 infection was simulated by treating worms with the receptor-binding domain (RBD) of the spike protein, which caused dose-dependent and time-dependent pharyngeal impairment in ACE2 worms but not in WT worms. The toxicity of RBD was prevented by administering an anti-human ACE2 antibody, demonstrating that interactions with the ACE2 receptor are essential. The ACE2-expressing worm strain was further used for pharmacological research with Raloxifene. In vitro, 1–3 μM of Raloxifene reduced the entry of lentiviral particles carrying the Wuhan variant and B.1.1.7 UK and B.1.1.529 Omicron strains into HEK293-ACE2, in addition to particles expressing N501Y-mutated or P681H-mutated spike proteins. Raloxifene (0.1–1 μM) completely counteracted RBD toxicity in ACE2 worms, indicating that this strain offers a cost-effective in vivo screening platform for molecules with effects involving interactions with the ACE2 receptor.

## 1. Introduction

The negative experience of the Coronavirus Disease 2019 (COVID-19) pandemic—with over 7 million deaths and 778 million infections worldwide [1]—has highlighted how the emergence of a new infectious agent in a globalized society causes devastating effects, underscoring how crucial it is to be prepared for new infections. At the same time, important lessons were learned about detecting and isolating the emerging pathogen, quickly understanding its effects, and testing candidate prophylactic and therapeutic strategies. Significant efforts were dedicated to studying the transmission, pathogenesis, and medical countermeasures of SARS-CoV-2. For this purpose, different vertebrate animals were used, including mice, non-human primates, hamsters, and ferrets [2]. However, pharmacological studies were hindered by strict national and international laws regulating animal experimentation—especially in infection studies requiring high biosafety levels—and the high costs associated with using vertebrate animals. Developing a cost-effective and more practical model could be very helpful in overcoming these limitations.

In this context, the invertebrate *Caenorhabditis* (*C.*) *elegans* offers several advantages, such as easy cultivation in the laboratory, low maintenance costs, and a short lifespan. These features make it a valuable model for in vivo preclinical pharmacological screening before testing drug candidates in vertebrate studies. This nematode has recently been used to study some bacterial and viral infections [3]. It has developed several responses to pathogens in order to survive and reproduce, but unlike higher eukaryotes, it does not show adaptive immunity. However, it was recently demonstrated that *C. elegans* can employ specific defense strategies reminiscent of the adaptive immune responses activated in higher eukaryotes and mammals. This renders it a good model organism for exploring the evolutionarily conserved pathways critical to immunity. This response is often triggered within cells by detecting infection-induced damage, mainly in the intestine or epidermis [4]. When encountering pathogenic microorganisms, *C. elegans* activates protective mechanisms, including an avoidance behavior, by detecting specific microbial molecules [5]. When a pathogen cannot be avoided, the nematode can mount an innate immune response by activating specific signaling pathways, producing and releasing defense molecules [6]. For example, *C. elegans* can detect a specific pathogen from the presence of a compound produced explicitly by that pathogen or a secondary product created by the host following infection. It is also able to transfer infection information to its progeny, partly through the RNAi pathway, thus protecting its offspring against pathogens.

This study aims to characterize a new transgenic *C. elegans* strain that constitutively expresses the human angiotensin-converting enzyme 2 (hACE2) receptor. Although *C. elegans* has an ortholog of the human ACE2 gene called *acn-1*, which encodes for an ACE-like protein with metallopeptidase activity, this protein has functions unrelated to human ACE2. The ACN-1 protein is required for larval development and adult morphogenesis and is hypothesized to be involved in larval seam cell fusion [4,5,6].

It is well known that SARS-CoV-2 infection, in addition to significantly impacting lung function, can cause gastrointestinal symptoms. In fact, the virus can interact with ACE2, which is expressed in large quantities in the intestinal tract and liver, thus causing symptoms such as vomiting, diarrhea, and abdominal pain [7,8]. To mimic the interaction of SARS-CoV-2 with intestinal cells and investigate the use of ACE2-expressing *C. elegans* as a model for quick and cheap pre-clinical studies, worms were administered with the receptor-binding domain (RBD) of the spike protein. This is also based on the knowledge that one of the main routes of infection in *C. elegans* occurs upon ingestion through the pharynx, resulting in intestinal colonization [9]. The onset of a specific RBD-induced toxic effect was assessed by scoring the worms’ pharyngeal motility and locomotion. This is because *C. elegans* can recognize human proteins with biologically relevant properties by developing specific dysfunctions in the pharynx or neuromuscular system [10].

To investigate the possible use of this model for pharmacological approaches, as a prototype drug, we employed Raloxifene, a compound proposed as an effective and safe anti-COVID-19 treatment. Although it is a second-generation selective estrogen receptor modulator clinically used for treating and preventing post-menopausal osteoporosis and cancer, Raloxifene exerts antiviral activity against pathogens such as influenza A, Ebola, and hepatitis C [11]. In addition, from the screening of 400,000 candidates in compound libraries conducted by the Exscalate4CoV consortium within the European Commission’s Horizon 2020 program, Raloxifene was determined to be the most promising drug based on its ability to regulate SARS-CoV-2 replication and reduce pro-inflammatory cytokines [12,13]. Data obtained from a small randomized controlled multicentre clinical trial with patients who had mild to moderate symptoms suggested a possible capability of the drug to reduce viral loads, limiting diffusiveness and contagiousness within the population. The proportion of participants with undetectable SARS-CoV-2 was higher in subjects treated with 60 or 120 mg/day of Raloxifene for seven days compared to the placebo group [13]. In vitro experiments on Vero E6 and Calu-3 cells showed that Raloxifene exerts antiviral activity, blocking SARS-CoV-2 replication [14]. Moreover, surface plasmon resonance studies from our group have recently reported that this compound can directly bind, although with low affinity, to the spike protein of SARS-CoV-2, its subunit 1, and the RBD [14]. However, it cannot be excluded that Raloxifene can directly influence viral entry machinery in other experimental conditions, possibly modulating host response.

Before testing the effect of Raloxifene in transgenic *C. elegans*, in vitro studies were conducted in human embryonic kidney 293-T cells stably expressing hACE2 (HEK293-ACE2) and pseudo-infected with lentiviral vectors expressing different spike variants on the envelope. Overall, the results obtained indicate that the new transgenic *C. elegans* strain expressing the human ACE2 represents a good experimental approach for modeling SARS-CoV-2 infection and that Raloxifene can affect the interaction between SARS-CoV-2 and ACE2, inhibiting viral entry. Although interesting, the information obtained in *C. elegans* must be validated in appropriate vertebrate models before drugs and vaccines are tested in human clinical trials.

## 2. Materials and Methods

### 2.1. C. elegans

Bristol N2 nematodes were obtained from the Caenorhabditis Genetics Center (CGC, Minneapolis, MN, USA) and labeled as wild-type (WT). The transgenic *C. elegans* strain expressing the hACE2 sequence fused with an mCherry reporter gene at the C-terminus, under the control of the Vacuolar H ATPase (vha)-6 promoter (*vha6*::ACE2::mCherry), was acquired from In Vivo Biosystems (Eugene, OR, USA) and designated as ACE2. Both *C. elegans* strains were cultured and maintained using standard breeding conditions. Experiments were conducted at 20 °C on standard Nematode Growth Media (NGM) seeded with *Escherichia coli (E. coli)* OP50 as food (CGC).

### 2.2. Brood Size and Larval Development

The impact of ACE2 expression on reproduction and larval development was determined in WT- and ACE2-synchronized worms by singly plating them at the L4 larval stage on NGM-agar plates seeded with *E. coli* OP50. On the following day, the number of eggs laid by each worm was recorded daily until egg-laying ceased. Larval development, under the same experimental conditions, was monitored daily, and the number of individuals at each larval stage was scored until they reached the adult stage.

### 2.3. Pharyngeal Behavior and Motility

To evaluate the effect of hACE2 expression, pharyngeal function and motility were assessed in synchronized WT and ACE2 worms at day 1 of adulthood. Only worms crawling on the bacteria were considered for the assays. Pharyngeal function was measured by counting the number of times the terminal bulb of the pharynx contracted per 1 min (pumps/min) [15]. To measure the worms’ motility, they were picked up and transferred into a well of a 96-well plate containing 100 μL of 10 mM phosphate-buffered saline (PBS) at a pH of 7.4. The body bend assay was scored by counting the number of left–right movements per 1 min (body bends/min).

WT and ACE2 nematodes were exposed to H_2_O_2_ to evaluate the sensitivity to chemical stressors. Briefly, L4 larvae were collected, washed with an M9 buffer to eliminate bacteria, and then exposed to 0.5 mM of H_2_O_2_ in 10 mM of PBS, pH 7.4, for 2 h (100 worms/100 µL) or to the same volume of 10 mM PBS, pH 7.4, alone (vehicle) as a control. The worms were plated onto NGM plates seeded with *E. coli* OP50, and pharyngeal pumping was scored 24 h later.

### 2.4. Lifespan and Health Span

The lifespan and health span of the WT and ACE2 worms were evaluated at 20 °C, maintaining the nematodes on standard NGM seeded with *E. coli* OP50. The nematodes were synchronized by egg-laying and transferred to fresh NGM plates daily during the fertile period to avoid overlapping generations. Dead, alive, and censored animals were scored during the transferring process. The animals were considered dead if they showed no movement, no response to manual stimulation with a platinum wire, and no pharyngeal pumping activity. Animals with exploded vulvas or those desiccated on the wall were censored [16]. The number of active movements was also assessed in nematodes employed for the lifespan assay to determine healthy aging. Animals crawling spontaneously or after manual stimulation were considered moving, while dead animals and animals not exhibiting crawling behavior were considered not moving.

### 2.5. Exposure of C. elegans to RBD

To mimic SARS-CoV-2 infection, WT and ACE2 transgenic worms were fed with the RBD of SARS-CoV-2 from the Wuhan strain. Nematodes were synchronized via egg-laying, and 48 h later, L4 larvae were collected from the plates and washed three times with 10 mM PBS, pH 7.4, to remove bacteria. Worms were incubated with 0.00001–1000 ng/mL of SARS-CoV-2 RBD diluted in 10 mM PBS, pH 7.4 (Trenzyme, Konstanz, Germany) (100 worms/100 µL) for 2 h at 20 °C on an orbital shaker and then plated on NMG plates seeded with *E. coli* OP50. Control worms were incubated with 10 mM PBS, pH 7.4 (vehicle). The pharyngeal pumping rate and the body bends were scored 2 and 24 h later.

To determine if the toxic effect of RBD administration was explicitly due to the binding of RBD to the hACE2 receptors expressed in transgenic nematodes, the anti-human ACE2 antibody (anti-hACE2, Santa Cruz Biotechnology, Dallas, TX, USA) was diluted at 1:200 (vol/vol) in 10 mM PBS, pH 7.4, and incubated for 1 h at room temperature with 100 ng/mL RBD before administration to the worms (100 worms/100 μL). In addition, anti-hACE2—diluted as previously described—was inactivated at 100 °C for 15 min and incubated with 100 ng/mL RBD for 1 h at room temperature before administration to the worms (100 worms/100 μL). Control worms were fed with the anti-hACE2 antibody diluted at 1:200 (vol/vol) in 10 mM PBS, pH 7.4, or 10 mM PBS, pH 7.4, only (vehicle). After 2-h incubation with orbital shaking, worms were transferred onto NGM plates seeded with *E. coli* OP50, and pharyngeal motility was assessed 2 and 24 h later.

### 2.6. Cells

The human embryonic kidney 293 (HEK293) cell line was obtained from Merck KGaA (Darmstadt, Germany; cod. # 85120602-CDNA). The HEK293 cells stably expressing human receptor ACE2 (HEK293-ACE2) were kindly provided by Prof. E. Biasini (Department of Cellular, Computational & Integrative Biology, University of Trento, Italy) [17]. Both cell lines were maintained in Dulbecco’s Modified Eagle Medium (DMEM; Euroclone S.p.A., Pero, Milan, Italy; cod. ECB7501L) containing 10% heat-inactivated fetal bovine serum (FBS, Gibco, Thermo Fisher, Segrate, Milan, Italy; cod. 10270), L-glutamine (Gibco; cod. 25030-024), non-essential amino acids (Euroclone; cod. ECB3054D), and penicillin/streptomycin (Corning, New York, NY, USA; cod. 20-002-Cl). HEK293-ACE2 required puromycin (Genespin, Milano, Italy). Cells were cultured in T25 flasks at 37 °C in a humidified 5% CO_2_ and routinely split every 4–5 days. The cells used in this study had not been passaged more than 20 times from the original stock.

### 2.7. Cell Viability

HEK293-ACE2 cells were seeded (2 × 10^4^ cells/well) on 96-well plates in a complete DMEM medium with 10% FBS. After incubation for 24 h at 37 °C in humidified 5% CO_2_, the medium was replaced with a fresh one containing Raloxifene (Dompé farmaceutici S.p.A., Milano, Italy) that was previously dissolved at 10 mM in dimethyl sulfoxide (DMSO) and diluted in DMEM at 1–30 µM. Control cells were treated with an equivalent DMSO concentration (vehicle). Cells were incubated for 24 h at 37 °C in humidified 5% CO_2_, and the medium was replaced with a fresh one without Raloxifene. After an additional incubation of 24 h at 37 °C in humidified 5% CO_2_, HEK293-ACE2 cells were treated with 5 mg/mL of 3-(4,5-dimethylthiazol-2-yl)-2,5 2,5-diphenyltetrazolium bromide (MTT; Sigma Aldrich, St. Louis, MO, USA) in 10 mM PBS, pH 7.4. After incubation for 4 h at 37 °C, the MTT was removed, and the cells were resuspended in isopropanol containing 0.04 M HCl. The absorbance of the samples was determined at 560 nm using a spectrophotometer (Infinite M200, Tecan, Männedorf, Switzerland), and the cell viability was expressed as a percentage of vehicle-treated cells.

### 2.8. Transduction Assay

HEK293-ACE2 and HEK293 cells were seeded (2 × 10^4^ cells/well) on 96-well plates in a complete DMEM medium with 10% FBS. To evaluate the effect of Raloxifene on the early stage of the viral infection, after 24 h at 37 °C in humidified 5% CO_2,_ the medium was replaced with fresh medium containing Raloxifene, previously dissolved at 10 mM in DMSO and diluted in DMEM at 1 or 3 µM. Control cells were treated with an equivalent DMSO concentration (vehicle). The cells were then incubated for 4 h at 37 °C in humidified 5% CO_2_ and then infected in the presence of 10 µg/mL of Polybrene (VectorBuilder, Chicago, IL, USA), with 5–50 MOI lentiviral vectors exposing the SARS-CoV-2 spike protein as surface glycoproteins in the Wuhan, B.1.1.7 UK, B.1.351 SA, N501Y, or P681H variant with eGFP as a gene reporter or the B.1.1.529 Omicron variant with eRFP as a gene reporter (VectorBuilder, Chicago, IL, USA). HEK293 cells were infected with lentiviral vectors as negative controls. Non-infected and non-drug-treated cells were employed as additional controls. The medium was replaced with a fresh one the day after the transduction. After a further 24 h of incubation at 37 °C in humidified 5% CO_2_, transduction efficiency was verified by determining the percentage of cells expressing GFP or RFP using a ZOETM fluorescent cell imager (Bio-Rad, Hercules, CA, USA). The ZOETM images were analyzed with Fiji software, an open-source platform for biological image analysis [17]. Transduction efficiency was expressed as the percentage of cells positive for GFP- or RFP-fluorescent signals.

### 2.9. Raloxifene Administration to C. elegans

Raloxifene was dissolved in DMSO at a concentration of 10 mM; diluted to 0.001–5 µM in 10 mM of PBS, pH 7.4; and incubated for 1 h at room temperature with 100 ng/mL of RBD before being administered in WT and ACE2 worms (100 worms/100 μL). Control worms were fed 100 μL of Raloxifene or 10 mM PBS, pH 7.4 (vehicle). The pharyngeal pumping rate, measured by counting the number of times the terminal bulb of the pharynx contracted over a 1-min interval (pumps/min), was scored 2 and 24 h later.

### 2.10. Western Blot Analysis

HEK293-ACE2 cells—treated for 3, 6, and 24 h with 3 µM Raloxifene or a corresponding volume of DMSO diluted in DMEM (vehicle)—were lysed for 15 min at 4 °C with 20 mM of Tris-HCl, pH 7.5, containing 150 mM of NaCl, 1 mM of Na_2_EDTA, 1 mM of EGTA, 1% NP-40, 1% sodium deoxycholate, 2.5 mM of sodium pyrophosphate, 1 mM of β-glycerophosphate, 1 mM of Na_3_VO4, and 1 µg/mL of leupeptin. Samples were centrifuged for 10 min at 16,100× *g*, and the protein content was quantified using a Pierce BCA Protein Assay Kit (Thermo Fisher Scientific Inc., Rockford, IL, USA). In total, 20 µg of total protein was loaded in each lane.

Protein extracts were also prepared from *C. elegans* using the following protocol. ACE2 and WT nematodes were synchronized via egg-laying and cultured at 20 °C on NGM plates seeded with *E. coli* OP50 as food. Worms were collected on the first day of adulthood (~100 worms) with an M9 buffer and washed to eliminate bacteria. Pellets were resuspended in 300 μL of 10 mM PBS, pH 7.4, and supplemented with protease inhibitor cocktail (Millipore, Milan, Italy). The samples were sonicated at 4 °C using the Bioruptor^®^ sonicator device (Diagenode, SA, Ougreé, Belgium): 30 s/30 s on-and-off intervals for 15 min at maximum energy. After centrifuging the samples at 15,700× *g* for 5 min at 4 °C, the supernatants were transferred to a clean 1.5 mL tube, and the protein concentration was determined using the Bradford Assay (Bio-Rad). In total, 25 μg of total protein was loaded in each lane. In addition, 20 ng of recombinant hACE2 protein (AdipoGen Life Sciences, Fuellinsdorf, Switzerland) was loaded as a control.

Proteins were separated via 10% SDS-PAGE and blotted onto a PVDF membrane (Millipore). To minimize background staining due to the non-specific membrane-binding of the antibody, the membranes were directly blocked with a blocking buffer (5% (*w*/*v*) comprising non-fat dry milk powder and 2% (*w*/*v*) bovine serum albumin in Tris-buffered saline with 0.15% Tween-20 (TBST) for 1 hour at room temperature. Then, they were probed with primary antibodies—anti-hACE2 mouse monoclonal antibody, clone AC18Z (1:1000, Millipore), or anti-β-actin mouse monoclonal antibody, clone C4 (1:2000, Sigma Aldrich)—at 4 °C overnight. After washing with TBST (10 minutes, three times), the membranes were incubated with a peroxidase-conjugated anti-mouse IgG secondary antibody (1:20,000, GE Healthcare, Milan, Italy) for 1 hour at room temperature. Chemiluminescence was detected via Clarity Max Western ECL Substrate Hybridization (Bio-Rad), and the membranes were scanned with a ChemiDoc XRS Touch Imaging System (Bio-Rad).

### 2.11. Statistical Analysis

Statistical analyses were performed using Prism GraphPad software v.10.2 (GraphPad Software, San Diego, CA, USA). All data points were included, except for experiments where negative and/or positive controls did not produce the expected outcome. An analysis of outliers was performed. The normal distribution of data was determined by applying the D’Agostino–Pearson, Anderson–Darling, Shapiro–Wilk, and Kolmogorov–Smirnov tests. The non-parametric Kruskal–Wallis test was employed to analyze data for which the normal distribution was not determined. Data with normal distribution were analyzed using an unpaired *t*-test and one-way or two-way ANOVA, corrected by a Bonferroni post hoc test. The results were expressed as means ± SD or ±SEM. A *p*-value of less than 0.05 was considered significant. For lifespan and health span studies, the number of dead and censored animals was used for survival analyses in OASIS 2 [18]. The *p*-values were calculated using the log-rank and Bonferroni’s post hoc tests between the pooled populations of animals.

## 3. Results

### 3.1. The Expression of hACE2 in C. elegans Affects Reproduction and Pharyngeal Function

**Figure 1:**
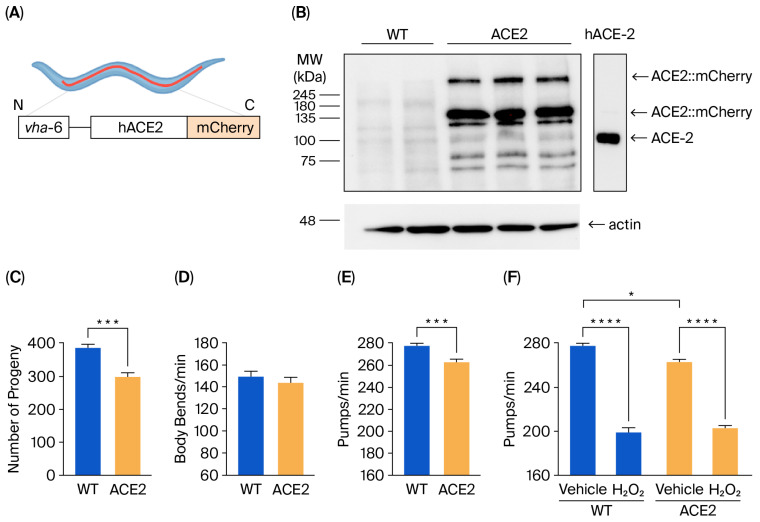
**Characterization of the transgenic *C. elegans* ACE2 strain.** (**A**) Schematic representation of the ACE2 transgenic *C. elegans* characterized in this study. The human ACE2::mCherry protein was mainly expressed in intestinal cells under the *vha-6* promoter to generate ACE2 transgenic *C. elegans*. (**B**) Western blots of transgenic ACE2 (ACE2) and wild-type (WT) worms (*n* = 3 biological replicates). An equal amount of proteins (25 µg) was loaded in each gel lane and immunoblotted with anti-human ACE2 or anti-actin antibodies. In total, 20 ng of recombinant human ACE2 (hACE-2) was analyzed as a positive control. (**C**) Broad size of WT and ACE2 worms. Data are the mean ± SEM (*n* = 7). *** *p* = 0.0003, Student’s *t*-test. (**D**) Motility and (**E**) pharyngeal activity of worms on the first day of adulthood. Data are expressed as the mean of (**D**) body bends/min (*n* = 10 worms/assay, 2 assays) or (**E**) pumps/min ± SEM (*n* = 12 worms/assay, 3 assays, **** *p =* 0.0001, Student’s *t*-test). (**F**) Sensitivity of worms to hydrogen peroxide. Worms were fed for 2 h with 0.5 mM of H_2_O_2_ in 10 mM of PBS, pH 7.4, or with the same volume of PBS alone (Vehicle). Pharyngeal pumping was scored 24 h after plating nematodes on NGM agar plates seeded with *E. Coli* OP50. Data are the mean of pumps/min ± SEM (*n* = 13 worms/assay, 4 assays). * *p* < 0.05 and **** *p* < 0.0001; Kruskal–Wallis test.

A new *C. elegans* strain expressing hACE2::mCherry under the *vha-6* promoter (Figure 1A) was characterized. The level of hACE2 protein expression was assessed via Western blot analysis of the lysates from WT and ACE2 worms on the first day of adulthood. Immunoreactive bands at approximately 135 kDa and 270 kDa, representing monomeric and dimeric forms of ACE2::mCherry, respectively, were detected in lysates of ACE2 worms but not in WT worms (Figure 1B). Some immunoreactive signals were also observed at a molecular weight lower than 135 kDa. Similar—although less intense—bands appeared in the lysates of WT worms and were absent in recombinant hACE2 proteins (Figure 1B). Based on these results, we concluded that the immunoreactive signals below 135 kDa likely result from the antibody against hACE2 binding to *C. elegans* proteins that are similar to the human ACE2 ortholog.

The impact of hACE2 expression on worm reproduction, development, feeding behavior, neuromuscular movement, lifespan, and health span was evaluated. A 22% reduction in the broad size, measured as the total number of progeny, was observed in ACE2 compared to WT (Figure 1C), indicating that hACE2 expression affected the worm’s reproduction. No effect was observed on the worms’ development, as indicated by the number of worms at different larval stages (from L3 up to the adult stage), which was similar between the ACE2 and WT nematodes (Figure S1). The neuromuscular function and the feeding behavior of adult worms were then evaluated by scoring the number of left–right movements in liquid per minute (body bends/min) and the function of the pharynx. No difference was observed between the movements of WT and ACE2 nematodes (Figure 1D), whereas there was a slight but significant 5% reduction in the pharyngeal pumping rate of ACE2 (Figure 1E). The ability of worms to react to oxidative stress was similar between the two strains, as demonstrated by the comparable reduction in pharyngeal pumping scored for WT and ACE2 nematodes after exposure to hydrogen peroxide (Figure 1F). Furthermore, the transgenic strain’s lifespan and health span were similar to the WT strain (Figure S2). In particular, the median lifespan was 20.3 ± 0.40 days and 19.9 ± 0.37 days for WT and ACE2 nematodes, respectively, and the median health span was 18.0 ± 0.32 days and 18.6 ± 0.37 days for WT and ACE2, respectively. These findings indicate that the expression of the hACE2 receptor in nematodes reduced their ability to reproduce and their pharyngeal function.

### 3.2. RBD Administration to ACE2 Worms Causes a Specific Pharyngeal Dysfunction

**Figure 2:**
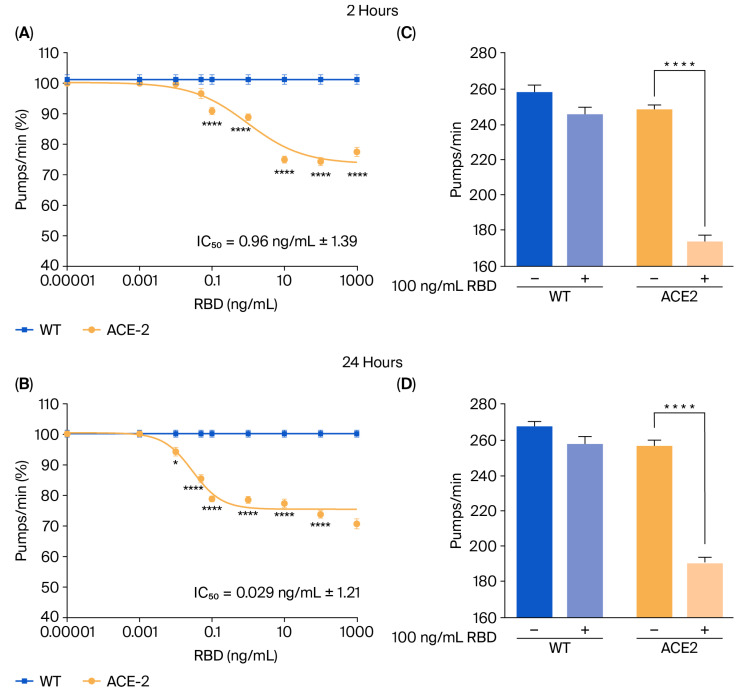
**RBD administration caused a specific toxic effect in ACE2 transgenic worms**. (**A**,**B**) Dose-dependent effect of RBD on the pharyngeal behavior of WT and ACE2 worms. Worms were fed for 2 h with increasing concentrations of RBD suspended in 10 mM PBS or the same volume of PBS alone as a control. Pharyngeal pumping was scored (**A**) 2 and (**B**) 24 h after plating nematodes on NGM agar plates seeded with *E. coli* OP50. Data are expressed as pumps/min (percentage of control) ± SEM (*n* = 10 worms/assay, 3 assays). * *p* < 0.05 and **** *p* < 0.0001 vs. WT treated with the corresponding concentration of RBD, one-way ANOVA, and Bonferroni’s post hoc test. (**C**,**D**) Effect of 100 ng/mL of RBD on the pharyngeal activity of WT and ACE2 worms scored (**C**) 2 and (**D**) 24 h after administration. Data are the mean of pumps/min ± SEM (*n* = 24 worms). **** *p* < 0.0001; Kruskal–Wallis test.

To mimic SARS-CoV-2 infection, ACE2 worms were fed with the RBD of SARS-CoV-2 from the Wuhan strain, and the onset of toxic effects was determined by evaluating the worms’ pharyngeal motility and locomotion 2 and 24 h after administration [10]. As shown in Figure 2A,B, RBD resulted in a dose-dependent pharyngeal impairment in ACE2 but not in WT worms. The pumping rate of ACE2 scored 2 h after the RBD administration was significantly reduced, starting at a concentration of 0.1 ng/mL and reaching the minimum value at 10 ng/mL (Figure 2A). At this time point, the half-maximal inhibitory concentration (IC_50_) value of RBD was 0.96 ng/mL ± 1.39. A thirty-three times lower IC_50_ value of 0.029 ng/mL ± 1.21 was measured 24 h after RBD administration (Figure 2B), indicating that it caused a worsening and permanent pharyngeal dysfunction. At 100 ng/mL, RBD resulted in 30.0% and 25.5% pharyngeal pumping inhibition in ACE2 worms 2 and 24 h after administration (Figure 2C,D). At this concentration, RBD did not affect the motility of ACE2 worms (Figure S3). Based on these results, the RBD concentration of 100 ng/mL was chosen for future experiments.

**Figure 3:**
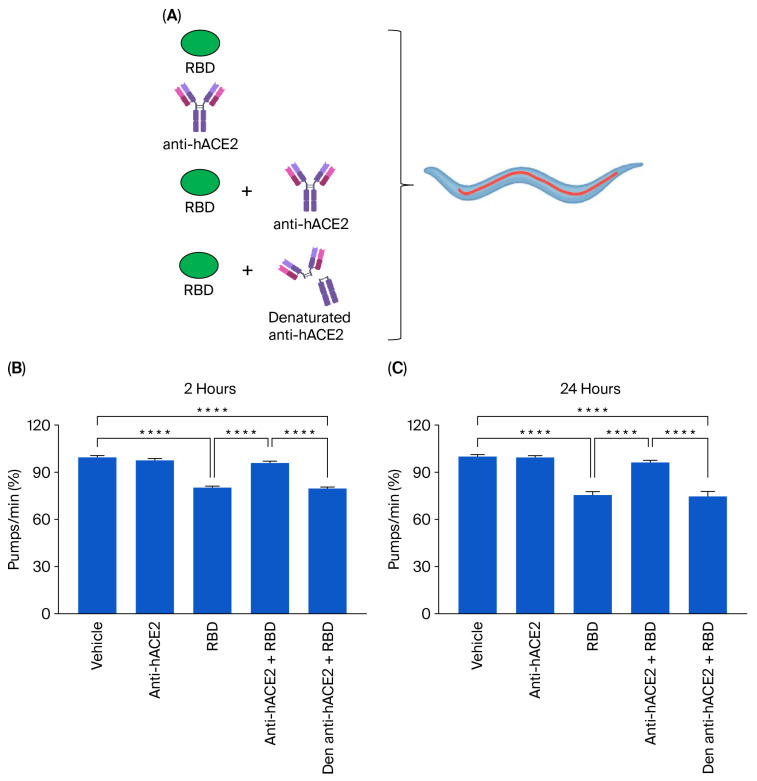
**Anti-hACE2 antibody counteracted the RBD toxic effect in ACE2 worms**. (**A**) Schematic representation of the treatment of worms with 100 ng/mL RBD alone or with the mouse monoclonal anti-human ACE2 antibody (anti-hACE2) (1:200 (vol/vol) RBD: anti-hACE2). The anti-hACE2 alone or 100 ng/mL of RBD, administered with the inactivated anti-hACE2 (denatured anti-hACE2), was administered as a negative control. (**B**,**C**) ACE2 worms were fed for 2 h with 100 ng/mL of RBD in the following: 10 mM PBS, pH 7.4, (RBD); 10 mM PBS, pH 7.4, alone (vehicle); anti-hACE2 antibody diluted in 10 mM PBS, pH 7.4 (1:200, vol/vol); 100 ng/mL RBD + anti-ACE2 antibody (anti-hACE2 + RBD); or 100 ng/mL RBD + denatured anti-hACE2 antibody (den anti-hACE2 + RBD). Pharyngeal pumping was scored (**B**) 2 and (**C**) 24 h after plating nematodes on NGM agar plates seeded with *E. coli* OP50. Data are the mean of pumps/min (% of vehicle-treated worms) ± SEM (*n* = 40 worms/assay, 4 assays). **** *p* < 0.0001; Kruskal–Wallis test.

To determine if the toxic effect of the RBD administration was specifically due to the interaction of RBD with the hACE2 receptors expressed in the transgenic nematodes, ACE2 worms were administered 100 ng/mL RBD in the presence or absence of anti-hACE2, and the pharyngeal pumping rate was scored 2 and 24 h later. Control worms were treated with 10 mM PBS, pH 7.4 (vehicle), or anti-hACE2 diluted in the vehicle (Figure 3A). The anti-hACE2 previously inactivated via incubation at 100 °C for 15 min was also administered as an additional control (Figure 3A). The anti-hACE2 alone did not affect the worms’ pharynx (Figure 3B,C), but when co-administered with RBD, it completely abolished RBD-induced pharyngeal toxicity (Figure 3B,C). These findings indicate that RBD causes a specific and permanent pharyngeal dysfunction in ACE2 nematodes mediated by the interaction with the ACE2 receptor. In addition, the pharyngeal dysfunction caused by RBD can be used as a readout of the mimicked infection.

### 3.3. Raloxifene Reduces the Entry of Lentiviral Particles Expressing SARS-CoV-2 Spike Variants In Vitro

**Figure 4:**
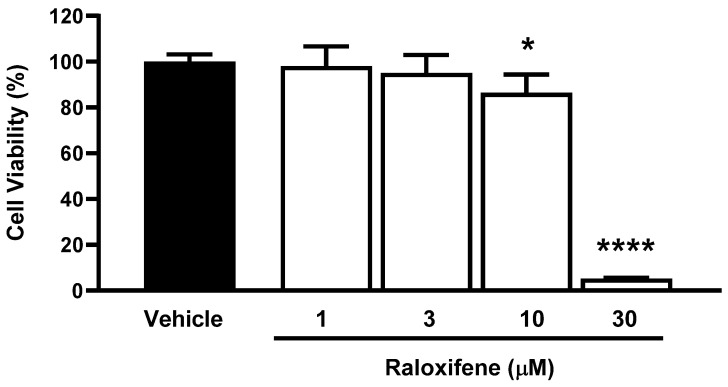
**Effect of Raloxifene on cell viability**. HEK293-ACE2 cells were treated with increasing concentrations of Raloxifene previously dissolved at 10 mM in DMSO and diluted in DMEM. Control cells were treated with an equivalent DMSO concentration (vehicle). Cell viability was evaluated 24 h after treatment using the MTT assay. Data are the mean ± SD of the percentage of viable cells compared to vehicle-treated cells (*n* = 5). * *p* < 0.05 and **** *p* < 0.0001 vs. vehicle according to one-way ANOVA and Bonferroni’s post hoc test.

Before using ACE2 worms for pharmacological studies with Raloxifene, we assessed the drug’s ability to reduce viral entry in vitro. We used HEK293-ACE2 cells and first evaluated the potential cytotoxic effects of Raloxifene to select drug concentrations that did not impair cell viability. After 24 h of exposure, Raloxifene significantly decreased the viability of HEK293-ACE2 cells starting at 10 µM, with maximal toxicity observed at 30 µM (Figure 4). Based on these findings, we chose Raloxifene concentrations of 1 and 3 µM for subsequent transduction experiments, where cells were infected with pseudotyped lentiviral vectors carrying different fluorochromes as reporter genes.

**Figure 5:**
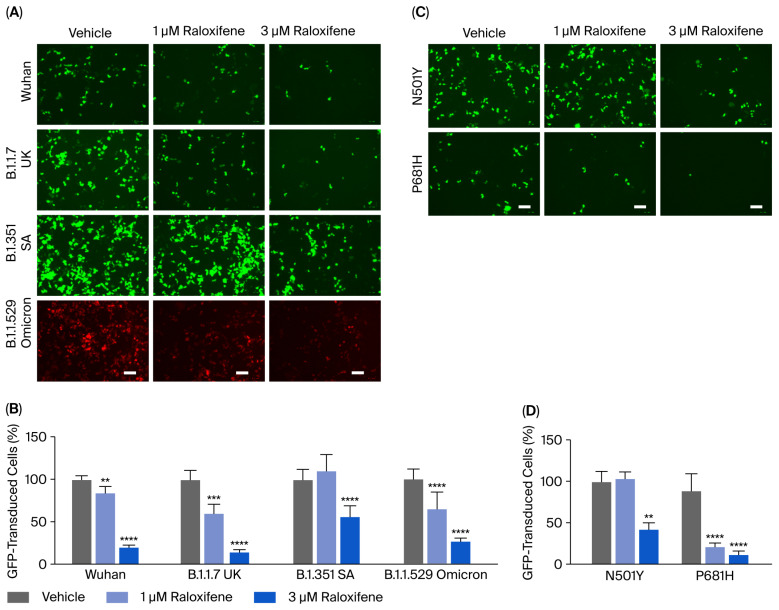
**Raloxifene reduced pseudoviral transduction in vitro.** (**A**,**B**) HEK293-ACE2 cells were pre-incubated for 4 h with 1 µM or 3 µM Raloxifene previously dissolved at 10 mM in DMSO and diluted in DMEM. Control cells were treated with an equivalent DMSO concentration (vehicle). Cells were then infected with pseudoviral particles, exposing them to the SARS-CoV-2 Wuhan, B.1.1.7 UK, B.1.351 SA, or B.1.1.529 Omicron spike variants. (**A**) Representative fluorescence microscopy images obtained. Scale bar, 100 µm. (**B**) GFP- and RFP-transduced HEK293-ACE2 cells expressed as a percentage of vehicle-treated cells. Data are the mean ± SD (*n* = 4–10, 3 independent experiments). ** *p* < 0.001, *** *p* < 0.0005, and **** *p* < 0.0001 vs. the corresponding vehicle according to one-way ANOVA and Bonferroni’s post hoc test. (**C**,**D**) HEK293-ACE2 cells were pre-incubated for 4 h with 1 µM or 3 µM Raloxifene previously dissolved at 10 mM in DMSO and diluted in DMEM. Control cells were treated with an equivalent DMSO concentration (vehicle). Cells were then infected with pseudoviral particles exposing the RBD-mutated N501Y or P681H-mutated spike variant. (**C**) Representative fluorescence microscopy images. Scale bar, 100 µm. (**D**) GFP-transduced HEK293-ACE2 cells expressed as a percentage of vehicle-treated cells. Data are the mean ± SD (*n* = 3–10, 3 independent experiments). ** *p* < 0.001 and **** *p* < 0.0001 vs. the corresponding vehicle according to one-way ANOVA and Bonferroni’s post hoc test.

HEK293-ACE2 cells were treated with Raloxifene previously dissolved at 10 mM in DMSO and diluted in DMEM, and then, they infected with lentivirus expressing the spike protein of SARS-CoV-2 in Wuhan, B.1.1.7 UK, B.1.351 SA, or B.1.1.529 Omicron variants. Control cells were transduced with an equivalent DMSO concentration (vehicle). In addition, HEK293 cells were infected with pseudoviral particles, exposing SARS-CoV-2 Wuhan or B.1.351 SA as additional negative controls. As already reported by our group, no transduction was observed in HEK293 cells not expressing hACE2 [17]. Vehicle-treated HEK293-ACE2 cells were efficiently infected with all the different lentiviral variants tested (Figure 5A). The lowest transduction capability was observed in cells pseudo-infected with the Wuhan variant, and the highest transduction was detected in B.1.1.529 Omicron variant (Figure 5A). Raloxifene significantly inhibited the transduction of HEK293-ACE2 cells in a concentration-dependent manner with all variants (Figure 5A,B). In total, 1 µM of Raloxifene reduced the percentage of transduction by 15%, 40%, and 36% in cells infected with the Wuhan variant and B.1.1.7 UK and B.1.1.529 Omicron isoforms, respectively (Figure 5B). In contrast, no effect was observed in cells treated with lentivirus expressing B.1.351 SA spike (Figure 5B). At 3 µM, Raloxifene reduced the percentage of transduced cells by 79%, 85%, and 73% in cells infected with the Wuhan, B.1.1.7 UK, and B.1.1.529 Omicron variants, respectively. In contrast, a significantly lower protective effect was observed in cells infected with the B.1.351 SA variant, in which the percentage of transduced cells was reduced by 43% (Figure 5B). These results indicate that Raloxifene can act on the viral pre-entry phase of the infection, counteracting the infection.

Additional experiments were performed using lentiviral particles displaying spike proteins carrying the RBD-mutated N501Y or the P681H single-point mutation present within the furin cleavage site region of the spike protein. We observed a higher transduction capability in cells pseudo-infected with the N501Y variant compared with the P681H variant (Figure 5C). Raloxifene only significantly inhibited the entry of pseudoviruses carrying the N501Y mutation by 57% at 3 µM (Figure 5C,D)—a lower extent compared to its ability to inhibit the entry of the Wuhan variant (Figure 5B,D). These data suggest that the N501Y mutation in RBD, known to determine a tight interaction between RBD and the hACE receptor [19], is relevant for the Raloxifene effect, confirming that its protection mechanism involves the interaction with hACE2. In cells transduced with lentivirus carrying the P681H spike substitution, Raloxifene was effective at 1 and 3 µM (Figure 5C,D), indicating that the presence of this mutation on the spike glycoprotein did not affect the drug’s protective activity. Altogether, these data firmly supported the capacity of Raloxifene to reduce the pseudovirus’s entry in vitro.

The exclusion of this biological outcome could be ascribed to an effect of Raloxifene on hACE2 expression, even in cells modified to overexpress the protein constitutively. Western blot analysis was performed on lysates of HEK293-ACE2 cells treated with the drug at 3 µM for 3 to 24 h. Raloxifene did not modify the expression of hACE2 (Figure S4), indicating that its ability to reduce the entry of the SARS-CoV-2 pseudovirus in vitro is not mediated by the modulation of this host cell receptor’s expression.

### 3.4. Raloxifene Protects ACE2 Transgenic Worms from the Toxic Effect of RBD

**Figure 6:**
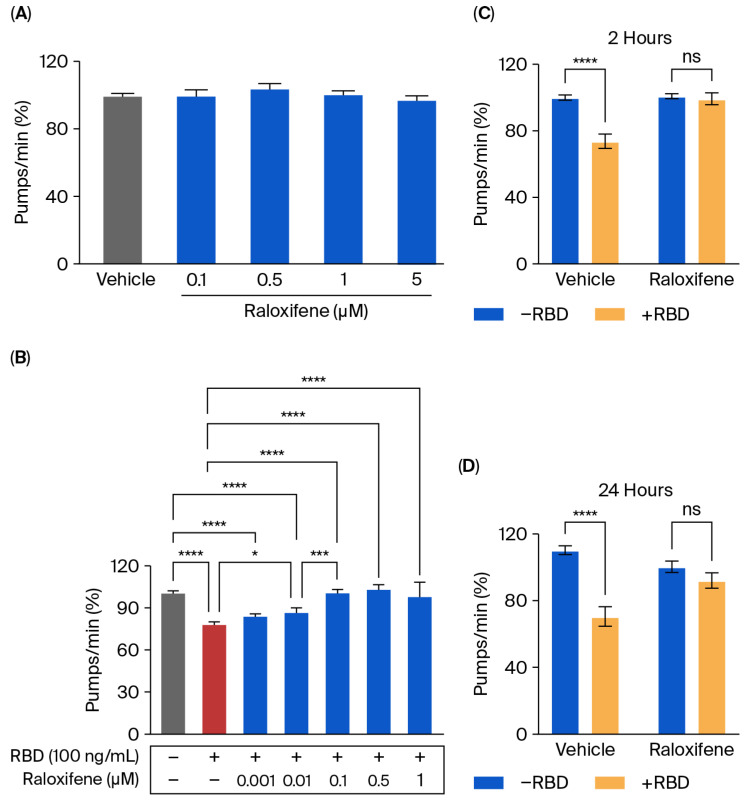
**Raloxifene protected ACE2 worms from the toxicity induced by RBD.** (**A**,**B**) ACE2 worms were fed for 2 h with (**A**) Raloxifene dissolved in DMSO at 10 mM and diluted to 0.1–5 μM in 10 mM PBS, pH 7.4, or with (**B**) 100 ng/mL RBD alone (red bar) or together with different concentrations of Raloxifene (blue bars). Control worms were fed the same volume of DMSO diluted in 10 mM PBS, pH 7.4 (vehicle, grey bar). Pharyngeal pumping was scored 24 h after plating nematodes on NGM agar plates seeded with *E. coli* OP50. Data are expressed as pumps/min (percentage of vehicle-treated worms) ± SEM (*n* = 20). * *p* < 0.05, *** *p* < 0.001, **** *p* < 0.0001, one-way ANOVA, and Bonferroni’s post hoc test. (**C**,**D**) ACE2 worms were fed for 2 h with Raloxifene dissolved in DMSO at 10 mM and diluted to 1 μM in 10 mM PBS, pH 7.4 (Raloxifene), or DMSO diluted in 10 mM PBS, pH 7.4 (vehicle), in the presence (+RBD, yellow bar) or absence (−RBD, blue bar) of 100 ng/mL RBD. Pharyngeal pumping was scored (**C**) 2 and (**D**) 24 h after plating nematodes on NGM agar plates seeded with *E. Coli* OP50. Data are expressed as pumps/min (percentage of vehicle-treated worms) ± SEM (*n* = 20). **** *p* < 0.0001; interaction RBD/Raloxifene *p* = 0.0003 and 0.0014 at 2 and 24 h, respectively, according to two-way ANOVA and Bonferroni’s post hoc test.

Transgenic *C. elegans* expressing ACE2 treated with RBD was employed as a model to evaluate the protective effect of Raloxifene in vivo. First, the possible toxic effect of Raloxifene was considered by administering transgenic worms with increasing drug concentrations (0.1–5 μM). As shown in Figure 6A, Raloxifene did not affect the physiological pharyngeal function of worms at any of the doses considered.

Raloxifene was then administered to worms exposed to 100 ng/mL RBD, and the pharyngeal function was scored 24 h later. Significant protection from the toxicity caused by RBD was observed starting from 0.01 μM Raloxifene, and a complete counteraction of pharynx dysfunction was reached at 0.1 μM (Figure 6B). At this concentration, Raloxifene, already after 2 h of administration, restored the defect in pharyngeal contraction induced by RBD, returning organ function to physiological levels (Figure 6C). Interestingly, the effect of Raloxifene persisted with time since its beneficial effect was still present 24 h after administration (Figure 6D). This protective effect could be ascribed to the ability of Raloxifene to bind to RBD, thus counteracting its interaction with hACE2 receptors.

## 4. Discussion

The nematode *C. elegans* is widely used to study the mechanisms behind various human diseases [20,21] and is an emerging model for exploring host–pathogen interactions. In nature, *C. elegans* lives in a microbially rich environment where it encounters different pathogenic microorganisms, including bacterial, viral, fungal, and oomycete pathogens [20,21]. It has been used as a model host and as a tool for studying the biology of various human bacterial infections and fungal pathogens, as well as for discovering new antimicrobial treatments [22,23,24,25,26].

In this study, we propose using a new transgenic *C. elegans* strain constitutively expressing the hACE2 treated with the SARS-CoV-2 RBD to mimic SARS-CoV-2 infection. The expression of hACE2 from the *vha6* promoter exhibited an almost ubiquitous presence in all the worm’s organs, including the pharynx and intestine, which are the primary food access routes. For the first time, we reported that the expression of hACE2 significantly impaired the reproductive capacity of worms, in addition to their feeding behavior.

When these worms were treated with RBD, we observed a specific, dose-dependent, and permanent pharyngeal dysfunction. This suggests that interactions with hACE2 can activate unusual mechanisms that sustain organ damage over time. The data from experiments using an anti-hACE2 antibody also demonstrated the link between pharyngeal dysfunction and the RBD/hACE2 interaction. Additionally, *C. elegans* avoided the orthologue of transmembrane serine protease 2, a protease essential for the entry pathway of SARS-CoV-2 [27], ruling out the possibility that the toxic effect of RBD is due to the activation of some intracellular proteolytic cleavages. The mechanism underlying the ability of the SARS-CoV-2 RBD to impair pharyngeal function still requires clarification. We can only hypothesize that *C. elegans* can recognize this virus protein as a potentially dangerous stimulus, thus activating protective behaviors to reduce its ingestion.

Although *C. elegans* only has innate immunity and lacks many features of the vertebrate innate immune system—such as cellular immunity, inflammasomes, and complement immunity—it can recognize viral replication products, adopting defense strategies evocative of vertebrates’ adaptive immune responses. Notably, this is the first evidence that, in addition to viral nucleic acids [28], a viral protein can induce the onset of specific defense mechanisms in *C. elegans*, such as the inhibition of feeding behavior. In addition, viral and fungal pathogens can activate an immunological intracellular response to pathogens resembling the vertebrate-specific type-I interferon response [28]. Thus, the transgenic *C. elegans* strain expressing hACE2 might represent a good model for rapidly investigating the effect of new coronavirus variants using hACE2 as a cell entry receptor. This nematode expresses ADM-4, an ortholog of human ADAM-17, which is upregulated by SARS-CoV-2, facilitating its entry into cells [29]. It has recently been reported that the infection of worms with *Klebsiella aerogenes* caused a significant upregulation of *adm-4* and a reduction in pharyngeal pumping [30]. Thus, it cannot be excluded that RBD administration to ACE2 worms could also affect *adm-4* expression and activate some signaling pathways involved in the regulation of innate immunity in *C. elegans.* Because of its ability to be genetically manipulated, *C. elegans* could represent a robust system for studying the defense mechanisms of non-professional immune cells in a whole-animal context.

Raloxifene was selected as a prototype drug for investigating the potential use of the ACE2 *C. elegans* strain for pharmacological studies. It was first tested on HEK293-ACE2 cells, a relevant experimental system for investigating the role of hACE2 as the cell entry receptor, before being validated for use with ACE2 worms for pharmacological purposes. Although this drug was selected in the EXSCALATE platform for its ability to bind relevant SARS-CoV-2 proteins, the data obtained from surface plasmon resonance experiments indicated that it did not interfere with ACE2-spike subunit 1 binding. The data obtained in HEK293-ACE2 cells indicated that administering Raloxifene before adding a pseudovirus can inhibit infection. Interestingly, Raloxifene inhibited the entry of lentiviruses expressing the spike proteins of different SARS-CoV-2 variants at concentrations of 1–3 µM, similarly to those reported to inhibit viral replication in vitro [14]. These findings suggested that this drug can act in vitro on processes that could be involved in spike–hACE2 interaction.

Raloxifene also effectively protected ACE2 worms from the toxicity caused by RBD administration. This is the first observation of this drug’s ability to interfere with the RBD engagement of hACE2 in vivo. Raloxifene has been reported to have pleiotropic effects involving multiple mechanisms and processes, including the downregulation of ADAM17 [29]. Additional studies are required to investigate whether Raloxifene can modulate the expression of *adm-4* in ACE2 worms and if this effect can be linked to its protective effect.

Taken together, these findings indicate that the transgenic *C. elegans* strain expressing hACE2 can represent an affordable approach for the in vivo screening of the effects of molecules mediated by the interaction with the hACE2 receptor. In addition, generating genetically modified *C. elegans* to express proteins relevant to vertebrate infection could help model the interaction with pathogens and validate the protective effect of molecules with antiviral activity quickly and inexpensively. However, it must be noted that although this study indicated that *C. elegans* can provide some helpful information for initial drug efficacy screening, this complementary approach cannot replace the use of appropriate vertebrate models before drugs and vaccines are tested in human clinical trials.

## Data Availability

This article and its Supplementary Materials contain all relevant data. All raw data have been deposited on zenodo.org and are available upon request at luisa.diomede@marionegri.it.

## Supplementary Materials

The following supporting information can be downloaded at https://www.mdpi.com/article/10.3390/v17101387/s1. Figure S1: Human ACE2 expression in *C. elegans* did not affect development; Figure S2: Human ACE2 expression in *C. elegans* did not affect the lifespan and health span; Figure S3: RBD administration did not modify the motility of worms; Figure S4: Raloxifene did not affect the expression of ACE2 in HEK293-ACE2 cells.

## Abbreviations

The following abbreviations are used in this manuscript:

COVID-19	Coronavirus Disease 2019
*C. elegans*	*Caenorhabditis elegans*
hACE2	Human angiotensin-converting enzyme 2
RBD	Receptor-Binding Domain
WT	Wild-type nematodes
HEK293-ACE2	human embryonic kidney 293-T cells stably expressing hACE2
ACE2	Transgenic *C. elegans* strain expressing the human ACE2
vha6	Vacuolar H ATPase-6
PBS	phosphate-buffered saline
*E. coli*	Escherichia coli
NGM	Nematode Growth Media
DMEM	Dulbecco Modified Eagle Medium
FBS	Fetal bovine serum
DMSO	Dimethyl sulfoxide
MTT	3-(4,5-dimethylthiazol-2-yl)-2,5 2,5-diphenyltetrazolium bromide
anti-hACE2	Anti-hACE2 mouse monoclonal antibody
