# Supplementary material for: SARS-CoV-2 Entry Can Be Mimicked in C. elegans Expressing Human ACE2: A New Tool for Pharmacological Studies
Source: Viruses. 2025 Oct 18;17(10):1387. doi: 10.3390/v17101387 (PMC12567688; doi:10.3390/v17101387)
Supplement: Supplementary file 1 [file viruses-17-01387-s001.zip › viruses-3908570-supplementary.pdf]

Supporting Information

SARS-CoV-2 entry can be mimicked in *C. elegans* expressing human ACE2: a new tool for pharmacological studies

Margherita Romeo [1$], Sara Baroni [1$], Maria Monica Barzago [1], Samuela Gambini [1], Ada De Luigi [1], Daniela Iaconis [2], Andrea Rosario Beccari[2], Maddalena Fratelli [1], and Luisa Diomede [1,*]

[1] Department of Molecular Biochemistry and Pharmacology, Istituto di Ricerche Farmacologiche Mario Negri IRCCS, Milano, Italy; margherita.romeo@marionegri.it; sara.baroni@marionegri.it; mariamonicabarzago@marionegri.it; gambinisamuela@gmail.com; adadeluigi@marionegri.it; maddalena.fratelli@marionegri.it

[2] EXSCALATE, Dompé Farmaceutici SpA, Via Tommaso De Amicis, 95, 80131, Napoli, Italy; Daniela.Iaconis@exscalate.eu; Andrea.Beccari@dompe.com

[$] These authors equally contributed to the work

* Correspondence: luisa.diomede@marionegri.it

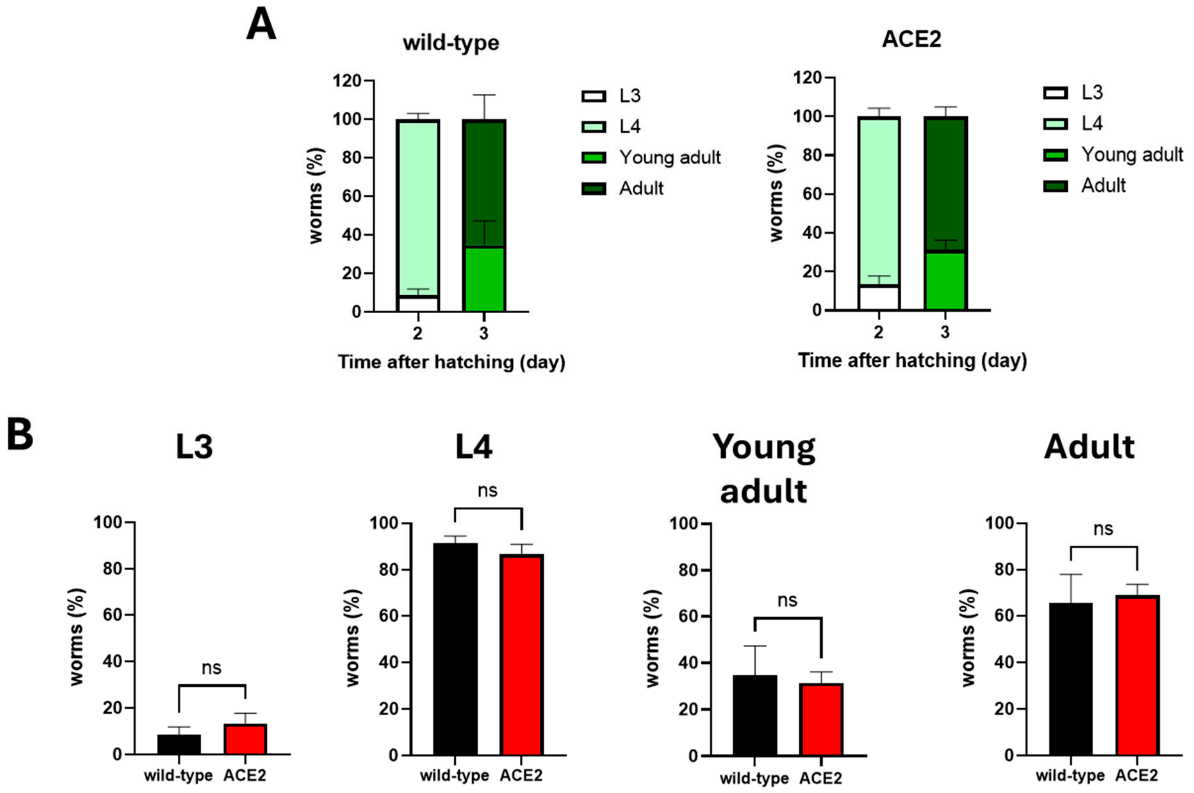

Supplementary Figure S1. **hACE2 expression in *C. elegans* did not affect the development**. (**A**) Larval growth of wild-type and ACE2 worms was rated at different times after eggs hatched to determine the number of nematodes at the L3 and L4 larval stages and the number of young adults and adults. Data are expressed as the percentage of the total worms ± SEM. (**B**) Percentage of worms at each larval stage.

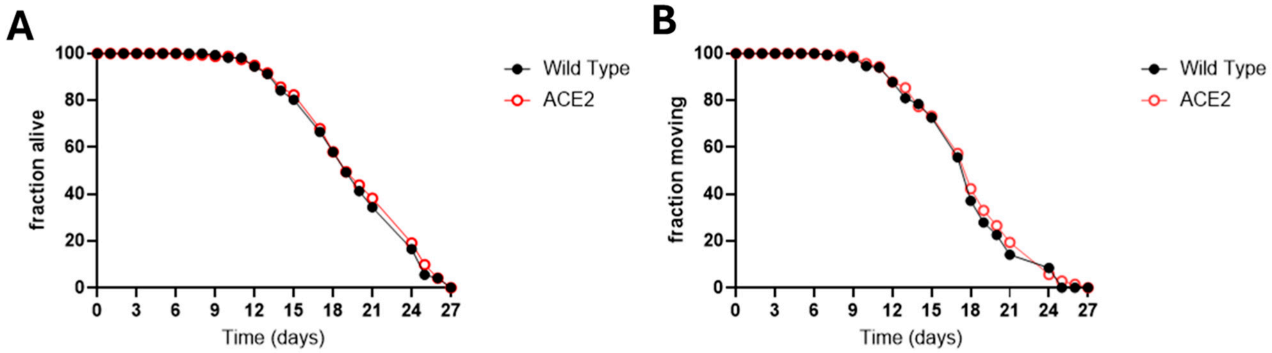

Supplementary Figure S2. hACE2 expression in *C. elegans* did not affect lifespan or health. (**A**) Lifespan and (**B)** health span of wild-type and ACE2 worms. Dead, alive, and censored animals were scored. Data are the mean ± SEM (N=180 worms from 3 independent experiments).

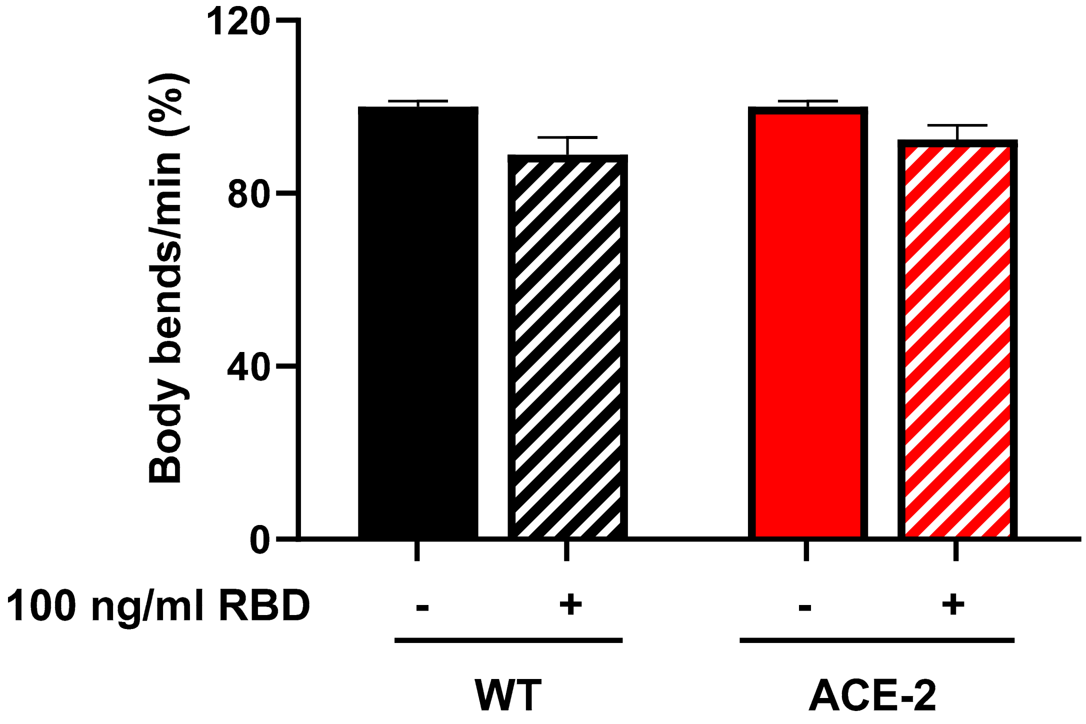

Supplementary Figure S3. **RBD administration did not modify the motility of worms.** Wild-type (WT) and ACE2 worms were fed for 2 hours with 100 ng/ml RBD suspended in 10 mM PBS or the same volume of PBS alone as a control. Body bends were scored 24 hours after plating nematodes on NGM agar plates seeded with *E. Coli* OP50. Data are the mean of body bends/min ± SEM (N = 20 worms).

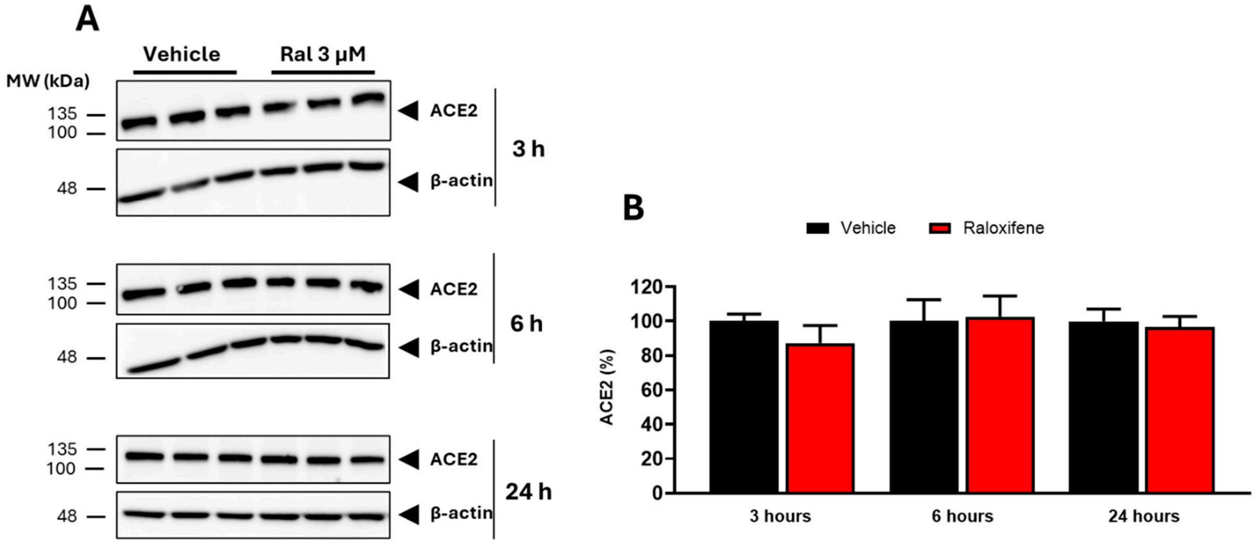

Supplementary Figure S4. **Raloxifene did not affect the expression of hACE2 in HEK293-ACE2 cells. (A)** Representative Western blot of hACE2 in lysates of HEK293-ACE2 cells treated for 3, 6, and 24 h with 3 μM Raloxifene or the corresponding volume of diluted DMSO (Vehicle). An equal amount of protein was loaded in each gel lane (20 μg) and immunoblotted with anti-hACE2 or anti-actin antibody. **(B)** hACE2 immunoreactive quantification is expressed as the percentage mean volume of the hACE2 band immunoreactivity/actin of the Vehicle-treated cells at the corresponding time point. Data are the mean ± SD (N=6 from 2 independent experiments).
